# Pyrosequencing analysis revealed complex endogenetic microorganism community from natural DongChong XiaCao and its microhabitat

**DOI:** 10.1186/s12866-016-0813-5

**Published:** 2016-08-26

**Authors:** Fei Xia, Yan Liu, Meng-Yuan Guo, Guang-Rong Shen, Juan Lin, Xuan-Wei Zhou

**Affiliations:** 1 Key Laboratory of Urban Agriculture (South) Ministry of Agriculture, and Engineering Research Center of Cell & Therapeutic Antibody (Ministry of Education), and School of Agriculture and Biology, Shanghai Jiao Tong University, No. 1-411# Agriculture and Biology Building, 800 Dongchuan Road, Shanghai, 200240 People’s Republic of China; 2 Department of Ecology and Evolutionary Biology, School of Life Sciences, Fudan University, E-401-8#, Life Science Building, 2005 Songhu Road, Shanghai, 200438 People’s Republic of China

**Keywords:** *Ophiocordyceps sinensis*, Pyrosequencing, Microorganism community, Community diversity, Microhabitats

## Abstract

**Background:**

*Ophiocordyceps sinensis* (DongChong XiaCao (DCXC) in Chinese), a fungal parasite of caterpillars, is a traditional Chinese medicine. Bioactive components isolated from natural DCXC possess a wide range of pharmacological actions. Many efforts have been directed towards isolating the fungi based on culture-dependent methods for investigation of fungal diversity in order to determine the anamorph of natural DCXC and find new medicinal fungi resources, and the results have been varied.

**Results:**

In the present study, a total of 44,588 bacterial and 51,584 fungal sequences corresponding to 11,694 and 9297 putative operational taxonomic units (OTU) were respectively identified by a Roche/454-based, high throughput sequence analysis of 16S rRNA genes and ITS regions. The main bacterial groups were Proteobacteria, Acidobacteria, Bacteroidetes, Actinobacteria and Firmicutes, while the Ascomycota, Basidiomycota and Zygomycota were the main fungal phyla. Proteobacteria presented 68.4, 49.5, 38.9 and 35.6 % of all bacteria in the sclerotia, stromata, external mycelial cortices and soil, respectively. As the main fungi phyla, Ascomycota presented 21.0, 45.6 26.4 and 59.3 % in the sclerotia, stromata, external mycelial cortices and soil, respectively. Bacterial and fungal communities were more diverse in the environmental sample than in the natural DCXC sample. Microbial communities were obviously distinct in each sample. Several novel unclassifiable bacterial (10.41 %) and fungal (37.92 %) species were also detected.

**Conclusions:**

This study revealed an abundant endogenetic fungal and bacterial resources and a variety of genetic information in natural DCXC by high-throughput 454 sequencing technology. Microorganism that had been discovered in natural DCXC will provide sources for screening the new bioactive metabolites and its biotechnological application.

**Electronic supplementary material:**

The online version of this article (doi:10.1186/s12866-016-0813-5) contains supplementary material, which is available to authorized users.

## Background

Natural *Ophiocordyceps sinensis* (syn. *Cordyceps sinensis*), known as DongChong XiaCao (DCXC, short for “natural DCXC” in following text) in Chinese and as the Chinese caterpillar fungus in western countries, is traditionally considered a parasitic complex of a fungus (*Hirsutella sinensis*) and caterpillars that belong to *Thitarodes*, *Hepialidae* and *Lepidoptera* [1]. It is endemic to alpine habitats on the Tibetan Plateau, located predominantly in Tibet and Tibetan autonomous prefectures of neighboring provinces and the high Himalayas [2]. Being described as an organism complex rather than a simple *O. sinensis* organism, the terms caterpillar fungus and natural DCXC are an entomophagous flask fungus in the new family Ophiocrdycipitaceae (Prenemycetes, Ascomycota) [3]. Isolated *O. sinensis* strain and DCXC have been used for academic research and processing products, however their names in concrete practices were often confused. Experimental methods in biochemistry have proved that DCXC consists of active constituents such as cordycepin, cordycepic acid, polysaccharides, nucleosides, ergosterol, peptides, aminophenol and trace elements [4]. The modern medical researches prove that natural DCXC and its active components possess a wide range of pharmacological actions, such as anti-inflammatory [5], antioxidant, antitumor, antihyperglycemic, antiapoptosis, immunomodulatory, nephroprotective and hepatoprotective [6]. Its immunoregulatory function plays an important role in anti-tumor effects, organ transplantation and the prevention of kidney, liver and heart disease [7]. Therefore, these myco-medicinal products, coupled with their associated mycelial cultures, are popular items in the traditional medicine market [8]. High commercial value and habitat degradation of natural DCXC has led to overexploitation, which has endangered the species in recent years. The number of natural DCXC populations is extremely limited, and its residential ecological environments are also potentially threatened [9]. To protect DCXC resources and its habitats, an effective approach is to study and develop substitutes represented by endogenetic microorganisms isolated from natural DCXC, based on consideration of its specific bioactive components. On the other hand, artificial cultivation of DCXC or artificial assistance of its growth may provide an alternative to protect this valuable resource [1]. In the past 30 years, scientists have been devoted to determine the anamorph of DCXC, and the investigation and isolation of the endogenetic fungi from natural DCXC [10–15]. Meanwhile, genetic diversity of natural DCXC has been estimated from a limited number of individuals or populations using various molecular methods [16–19]. Currently, the microorganism community composition, structure and functional activity of natural DCXC remain unclear.

With conserved and hypervariable sequence characteristics, the 16S rRNA genes and the internal transcribed spacer (ITS) regions were commonly used as the ideal marker sequence in the analysis of microorganism community diversity [20]. The 16S rRNA gene and ITS regions sequences permit the identification and prediction of phylogenetic relationships of prokaryotes and eukaryota [21, 22]. High-throughput sequencing, in particular pyrosequencing [23], has been applied widely to studies of microorganism community composition, structure and its ecology [24, 25]. Using this method, some trace microbes have been detected, and there have been new discoveries in the field of microbial population diversity in traditional Chinese medicinal materials (TCMM) [26], agriculture [27], marine ecology [28] and soil ecology [29]. According to traditional Chinese medicine theory, the geoherbalism formation of TCMM is based on many factors, among which the microenvironment of rhizosphere soil plays an important role in the formation of secondary metabolism in medicinal plants. Therefore, based on the diversity of microbial communities by the clone library approach [30], the natural DCXC couple with its microhabitat soil was divided into four parts: stroma, larva, external mycelial cortices and soil, to in each sample using high throughput pyrosequencing platform (Roche/454 GS-FLX Titanium System) and quantitative real-time PCR (qPCR) techniques. The aim of the study was to detect the microbial diversity and community structure of natural DCXC and its microhabitats. In subsequent, we analyzed the relationship between the microorganism community composition and structure in various specimens with regard to the medicinal components, fungal parasitisation of the larva, and growth and development. It lays a foundation for exploring the genetic diversity and functioning of DCXC microbiota as a model for further biotechnological developments.

## Results

### Community diversity

**Fig. 1:**
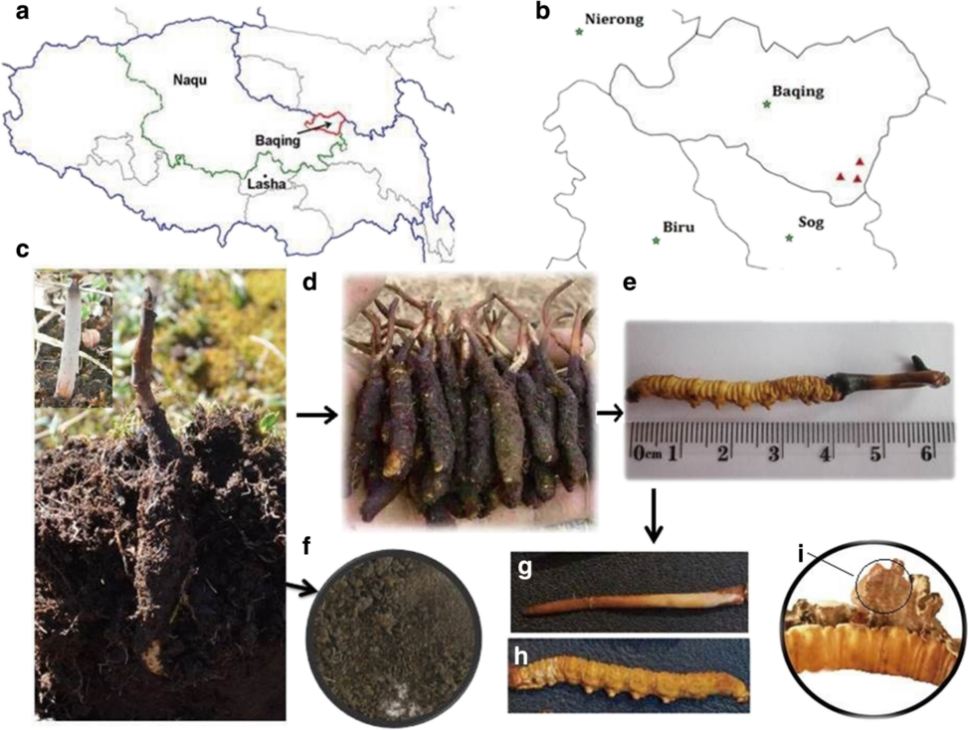
Collection and isolation of DCXC and its microhabitat samples. **a** The sample was collected from Ya-an ethnic township, Baqing country of Nagqu Prefecture; **b** The samples for this study were collected in three different populations at least 50–100 m apart at 4520 m above sea level; the *stars* indicated the governments of Biru, Nierong, Sog and Baqing county; the *triangles* indicated the sampling locations in the Ya-an ethnic township, Baqing County; **c** habitat of natural DCXC; **d**-**i** after collection of growing DCXC (**d** and **e**). The collected samples were divided into DCXC and its growth microhabitats. Microhabitat samples included soil adhering to the surface of the membrane (**f**), and external mycelial cortices (membrane covered around the larva) (**i**). The DCXC was divided into sclerotia (**g**) and stroma (**h**)

High quality sequences of 44,658 16S rRNA genes and 51,584 ITS reads were obtained with at least 9042 bacterial 16S rRNA gene and 8585 fungal ITS reads in each sample. The results indicated that a large number of bacterial and fungal microorganisms inhabiting the natural DCXC (sclerotia and stromata) (Fig. 1g, h) and its microhabitat (external mycelial cortices and soil adhering to the surface of the membrane covered DCXC) (Fig. 1i, f). Although we got nearly ten thousandsequences per sample for bacteria and fungus community, the slope of the rarefaction curve was still not flat at different similarity cutoff values, indicating that there were still microorganisms that was not detected (Additional file 1: Figure S1). The diversity and richness of bacterial and the fungal community of different natural DCXC samples and its microhabitat were represented by the indices (Table 1). The OTU (Operational taxonomic units) numbers and Shannon’s index of the soil sample were higher than the other samples, both in the bacterial and fungal communities (Table 1), which indicated a higher diversity of the bacterial and fungal community in the soil adhering to natural DCXC. This was also demonstrated by the curve of OTU Rank abundance (Additional file 2: Figure S2) and Simpson’s index (Table 1). Interestingly, the OTU number and the Shannon’s index showed that the bacterial community diversity was highest in the soil, followed by external mycelial cortices and stromata, and fewest in sclerotia at each similarity level. For the fungal community, the OTU number revealed the same trend as the bacteria; however, this trend in Shannon’s index was disturbed for external mycelial cortices and stromata.

**Table 1:** Summary of the diversity indices of different samples from DongChong XiaCao (DCXC; *Ophiocordyceps sinensis*) and its environmental samples

		Bacteria				Fungi			
Similarity	Sample names	OTU Numbers	Chao1 ^a^	Simpson	Shannon	OTU Numbers	Chao1	Simpson	Shannon
97 %	sclerotia ^b^	1982	3050.38	0.0069	6.23	1709	3638.45	0.0162	5.81
	stromata	2914	5409.71	0.0069	6.95	2109	4894.79	0.0120	5.68
	external mycelial cortices	4270	9127.36	0.0010	7.90	2450	6611.38	0.0200	5.60
	Soil	5809	13855.43	0.0003	8.31	3204	9554.88	0.0071	6.84
95 %	sclerotia	1447	2085.29	0.0097	5.75	1372	2855.18	0.0182	5.53
	stromata	2254	3711.34	0.0081	6.45	1639	3681.45	0.0154	5.35
	external mycelial cortices	3505	6743.64	0.0016	7.57	1998	4892.88	0.0210	5.40
	Soil	4912	10329.53	0.0005	8.05	2722	7445.69	0.0084	6.55
90 %	sclerotia	897	1188.80	0.0182	5.09	956	1926.36	0.0223	5.07
	stromata	1341	1984.05	0.0139	5.72	1039	1934.23	0.0181	4.92
	external mycelial cortices	2228	3577.16	0.0027	6.88	1391	2861.02	0.0253	5.03
	Soil	3216	5724.56	0.0012	7.38	2081	4787.90	0.0100	6.17

^a^ To calculate all the indices, the sequences number in each samples were normalized to the same, with 9042 bacterial 16S rRNA gene sequences and 8585 fungal ITS sequences

^b ^Abbreviation of the samples name: stroma (ZiZuo in Pinyin simplified as stromata), membrane covered around larva (JunPi in Pinyin simplified as external mycelial cortices), larva (ChongTi in Pinyin simplified as sclerotia) and the soil adhering to the surface of the membrane covered DCXC. The detailed image is shown in Fig. 1

Similarly, richness was highest in the soil sample, followed by external mycelial cortices and stromata. The least rich bacterial and fungal communities were in the sclerotia sample, which was in accordance with the Chao1 index (Table 1). The richness of microorganism community indicated that the soil and the external mycelial cortices in contact with the soil promoted microorganism growth.

### Community composition

**Fig. 2:**
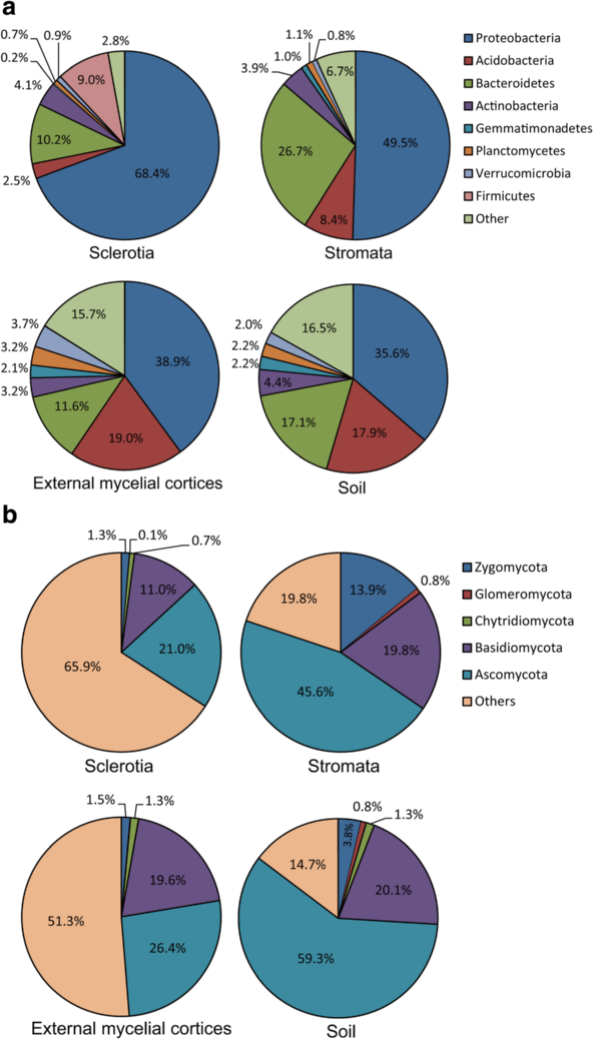
Bacterial and fungal community patterns of different samples. **a** bacterial community at the Phylum level; **b** fungal community at the Phylum level. The percentages on the vertical axis of the graph represent the proportion of each microbe group in the total obtained sequences for each sample

According to the taxonomy results of 16S rRNA gene and ITS sequences obtained by a localblast search against the Silva and UNITE databases, the microbial compositions in the different samples were analyzed (Fig. 2). The bacterial community composition was varied in different samples of natural DCXC and the microhabitat samples. Proteobacteria was the main group in different samples and presented 68.4, 49.5, 38.9 and 35.6 % of the total bacteria detected in sclerotia, stromata, external mycelial cortices and soil, respectively (Fig. 2a). The proportion of Acidobacteria in the sclerotia was rather small, representing only 2.5 %. In contrast, Acidobacteria in the other three samples were higher: 8.4 % in stromata, 19.0 % in external mycelial cortices and 17.9 % in the soil. Bacteroidetes was another notably abundant bacterial group, with the lowest proportion, 10.2 %, in sclerotia and the highest, 26.7 %, in stromata. In addition, Actinobacteria, Planctomycetes and Verrucomicrobia bacteria, as well as Firmicutes, were also detected in the samples, although at small fractions. Finally, we also detected notable numbers of unclassified bacteria, especially in the external mycelial cortices and soil samples, representing 15.7 and 16.5 % of the total sequences in these samples, respectively, indicating a higher diversity of unknown microorganisms in these two samples.

For the fungal community, we detected three main phyla in the samples from DCXC: Ascomycota, Basidiomycota and Zygomycota (Fig. 2b). Ascomycota was the most dominant group in the samples, representing 21.0 % in sclerotia, 45.6 % in stromata, 26.4 % in external mycelial cortices and 59.3 % in the soil. Basidiomycota, as the second most abundant fungal community group, was present at its lowest level, 11.0 %, in the sclerotia and was highest in the soil at 20.1 %. While in the stromata and external mycelial cortices, the portions of Basidiomycota fungi were 19.8 and 19.6 %, respectively. Zygomycota was present in low numbers in the sclerotia, external mycelial cortices and soil samples, but was rather higher, accounting for 13.9 % of the total detected fungi, in the stromata. The limitation of the resolution of the ITS sequence meant that there was a notable fraction of the ITS sequences in each sample that could not be classified exactly, especially in the sclerotia and external mycelial cortices, with 65.9 and 51.3 % of undefined sequences in these two samples, respectively. These results suggested an undiscovered abundant fungal community resource in the samples from natural DCXC.

The compositions of bacterial and fungal community were further analyzed at the genus level. The huge undiscovered microorganism resource in the natural DCXC and the resolution limit of the rRNA gene and the ITS region sequence, resulted in a large proportion of unclassified sequences at the genus level. For the bacterial community, undefined sequences accounted for 29.06 in the sclerotia, 54.63 in the stromata, 78.54 in the external mycelial cortices and 80.73 in the soil. These unclassified sequences suggested a huge bio-resource that may be related to the producing of medical materials in natural DCXC. Meanwhile, the most abundant genera detected in the sclerotia included *Pseudomonas* and *Rhodoferax*, with 9.24 and 9.11 % of the total sequences, respectively. While other genera, such as *Pedobacter* (3.42 %), *Sphingomonas* (2.29 %), *Collimonas* (2.78 %) were also detected. The main genera detected in the stromata were *Pedobacter* (8.05 %), *Rhodoferax* (6.91 %), *Variovorax* (4.41 %) and *Mucilaginibacter* (3.96 %). For the external mycelial cortices sample, the *Thermomonas* (3.43 %) and *Gemmatimonas* (2.13 %) were the main genera detected. In addition, *Ferruginibacter* (3.07 %), *Gemmatimonas* (2.22 %) and *Terrimonas* (1.93 %) were the main genera in the soil sample. Detailed information on the bacterial genera and their proportions detected in each sample is shown in Additional file 3: Table S1.

The undefined fungal ITS sequences also accounted for a significant percentage of the total sequences in the sclerotia (73.64 %), stromata (85.52 %), external mycelial cortices (87.55 %) and soil (67.32 %). The *Entoloma* were the main genus detected in the sclerotia (2.62 %) and external mycelial cortices (3.47 %) samples. It was also distributed in the soil sample, but the percentage was only 0.37 %. *Exophiala* (2.90 %), *Cladophialophora* (1.77 %) and *Phaeomollisia* (1.45 %), were the main genera detected in the sample stromata. *Tetracladium* (1.38 %), were the main genus in the external mycelial cortices sample. Finally, for the soil sample, *Cladophialophora* (3.30 %), *Verrucaria* (3.10 %), *Sebacina* (2.96 %) and *Gyoerffyella* (2.33 %) were the main genera detected. For more information of the fungal community genera and their proportions detected in each sample are shown in Additional file 4: Table S2.

### Community similarities in different samples

**Fig. 3:**
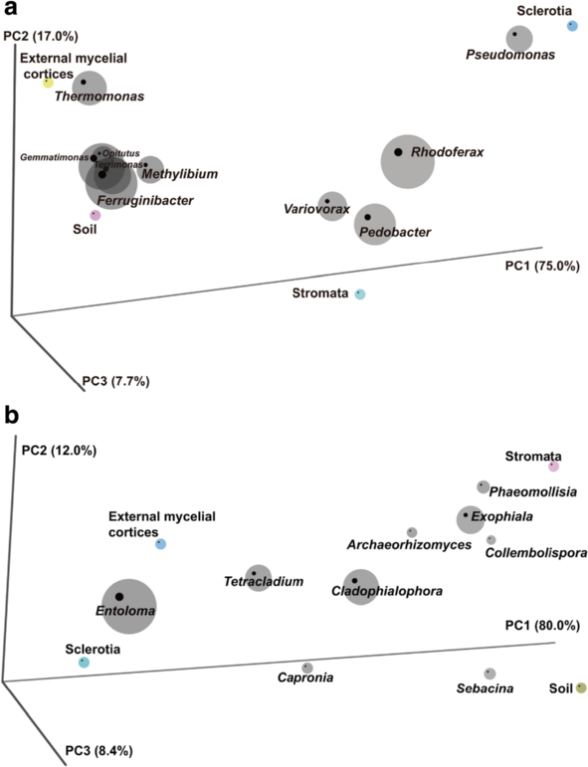
Principal coordinates analysis of bacterial and fungal communities in different samples of natural DCXC. Percentages on the axes of the graph represent the explained variance of total variance. The OTU data matrix used in the analyses was clustered at the 97 % similarity and the principal coordinates analysis was based on the jclass model. OTUs shown in the profiles were relatively more abundant ones. The circle in color indicated the four samples distributed in the ordination. The circle in gray indicated the OTUs that areas symbolize the abundant, and the different circles indicated the number of sequences contained in each OTU

The microorganism community similarity in different samples of natural DCXC was determined by weighted principal coordinates analysis (PCoA). The bacterial community in the soil sample appeared more similar to the bacterial community in the external mycelial cortices. These two samples clustered separately from the other two samples, sclerotia and stromata (Fig. 3a). This was also demonstrated by the unweighted pair group method with arithmetic averages (UPGMA) trees constructed from thetaYC distances, in which the bacterial communities of soil and external mycelial cortices were in the same group, while the samples stromata and sclerotia were in another group (Additional file 5: Figure S3-A). In addition, a Venn diagram showed the shared OTUs with 97 % similarity of the bacterial community in each samples from DCXC (Additional file 6: Figure S4-A). Soil and external mycelial cortices samples shared 1054 OTUs; meanwhile, that soil and stromata samples shared 877 OTUs, and there were only 406 OTUs shared by soil and sclerotia. This suggested that the bacterial communities in soil and external mycelial cortices were more similar, which was constant to the results of PCoA and the UPGMA clustering analysis. The defined bacteria at the genus level in the soil sample mainly were *Ferruginibacter* and *Gemmatimonas*, while the *Thermomonas* and *Pseudomonas* were the main genera in the sample external mycelial cortices and stromata, which was constant with the composition of the bacterial community at the genus level (Additional file 3: Table S1).

Fungal communities in the sclerotia and external mycelial cortices samples were more similar, with *Entoloma* being abundant in these two samples. Meanwhile, fungal communities in samples of soil and stromata were only distantly related to those in the sclerotia and external mycelial cortices (Fig. 3b). UPGMA trees constructed from a ThetaYC distances matrix showed that the fungal community structure in external mycelial cortices and sclerotia was more similar to each other than they were to those of the sample stromata and soil (Additional file 5: Figure S3-B). In addition, a Venn diagram (Additional file 6: Figure S4-B) showed that the shared OTUs with 97 % similarity of the fungal community in each samples from DCXC and its microhabitats, which indicated that external mycelial cortices and sclerotia shared 406 OTUs. Meanwhile, 344 OTUs were shared by external mycelial cortices and stromata, and 250 OTUs were shared by external mycelial cortices and soil. This demonstrated that a similar fungal community structure in the external mycelial cortices and sclerotia samples. Soil and stromata samples contained rather different fungal communities compared to the external mycelial cortices and sclerotia samples, suggesting a high diversity of fungi in the different samples of DCXC and its microhabitats. For the defined fungal genera in the samples, the levels of *Entoloma* provided a partial explanation for the distribution of sclerotia and external mycelial cortices, while other genera did not show a tendency to be present in a particular sample internal microenvironment, indicating that these fungi were disparate in the different part of DCXC.

### Abundance of total bacteria and fungi

**Fig. 4:**
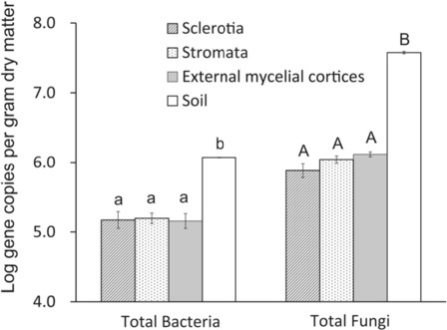
Abundance of the total bacterial and fungal community in different samples isolated from natural DCXC. The error bars indicate SDs (*n* = 3), with some error bars smaller than the symbol. Samples containing a different letter indicate that there were significant differences (*p* < 0.05). The unit of abundance was the log value of the gene copies per gram of dry materials. The moisture content of each sample was measured by gravimetric analysis after oven-drying (105 °C) of triplicate samples

The abundance of total bacterial and fungal communities was detected qPCR (quantitative real-time PCR) based on the SYBR Green method. The qPCR results indicated that the soil sample contained the most abundant bacterial and fungal community compared with the other samples from natural DCXC. The abundance of bacterial community were higher than the fungal community in natural DCXC samples (Fig. 4). Bacterial community abundance was 1.18 ± 0.01 × 10^6^ copies per gram dry materials in the soil sample, which was significant higher than in in the sclerotia, stromata and external mycelial cortices samples (*p* < 0.05). With the fungal abundance was 3.80 ± 0.15 × 10^7^ copies per gram dry material, abundance in the soil sample was significant higher than in in the other samples (*p* < 0.05). The results of a *T*-test indicated that the abundances of total bacterial and fungal communities were significantly higher in the soil sample than in the other samples (*p* < 0.05), and the abundance of the total fungal community was significant higher than the total bacterial community abundance in each sample (*p* < 0.05).

## Discussion

Natural DCXC is an expensive, endangered, traditional medicinal drug, which comprises many fungi and a larva. Since the 1980s, the separation and identification of the anamorph of DCXC have been the main focus in this field. More than 30 fungal strains belonging to 13 genera, which are involved in anamorphic types of the DCXC, have been reported in China. In recent years, the relationship between DCXC and its microhabitats has received more attention. Zhang et al. [31] investigated the mycobiota of natural DCXC using a traditional culture-dependent method. In total, 572 fungal cultures were isolated from different parts (including stromata, sclerotia, and external mycelial cortices) of natural DCXC. For the growth microhabitat of DCXC, Li et al. [32] analyzed soil fungal community structures in propagated DCXC by DGGE, and found that the ITS2 sequences of fungi in soil samples were highly similar to those of *Inocybe* (Fr.) Fr., *Tricholoma* (Fr.) Staude, *Entorrhiza* C.A. Weber, unconfirmed Ascomycota, and soil fungi in GenBank; the similarity of the soil fungal population structure was only 19.4 % ~ 50.1 %. Based on the above-mentioned analysis, at the technical level, the traditional culture-dependent method was used for primary separation of fungi from the DCXC. Modern bio-techniques were used for the identification of the DCXC anamorph in the isolates, rather than to investigate the microorganism community [33–35]. Many years of research have proved that *H. sinensis* is the anamorph of natural DCXC collected from different geographical regions [10, 36]. However, *H. sinensis* and *O. sinensis* belong to different stages of the life cycle of the same organism [12, 37]. *O. sinensis* pathogenicity has clearly been shown, but only by a few researchers. The aims of the previous studies were to determine the anamorph of DCXC that could produce similar metabolites to DCXC. Currently, *H. sinensis* mycelia have been applied successfully for in large-scale fermentation to produce various drugs and health products [1], which has led to limited protection of natural DCXC resources. Although *O. sinensis* is defined to be only one species, natural DCXC and its microhabitats contain a variety of microorganism [10], and form a complex host-microbiota-environment microecosystem. In this study, the pyrosequencing analysis reveals an unexpectedly high diversity of the microorganism community in the complex microecosystem. Analysis of composition and structure of microorganism communities benefits to unsolved problems in DCXC study, for example, how fungus infected the larvae, what was the relationship (symbiosis, parasitism or pythogenesis and etc.) of endophytic fungus and its host organism interaction. However, in this microecosystem, the relationships among microorganisms inhabiting in DCXC need to be further explored. The influence of these microorganisms to the reproductive and individual development of DCXC will also need further investigation. In addition, numerous of unidentified sequences were detected in the natural DCXC samples and its micro-environments. There are two possible reasons for explanation of this result. Firstly, the research targeting the micro-ecology in Tibetan Plateau was few all over the world. Secondly, scientists for the study of DCXC mainly came from China, and focused their interesting on isolation and identification of the fungus of the asexual generations (anamorph), and fermented these fungi by using artificial methods, etc. So there was a little piece of information on endogenetic microorganism from natural DCX in the database. The bacterial population of DCXC was only one report available [30], and the effects of bacteria on the growth process and medicinal composition of natural DCXC are unclear. Based on the results analysis of this study, the main groups of bacteria in the different samples were Proteobacteria, Acidobacteria, Bacteroidetes, Actinobacteria and Firmicutes. Among the bacterial communities, the Proteobacteria is a major group (phylum) of bacteria, which include a wide variety of pathogens. The results suggest that bacteria may be relative to pathopoiesis of the larva. Acidobacteria is widely distributed in nature and plays important roles in various ecosystems [38]. Seven genera of Acidobacteria were isolated and reported, and confirmed by Bergey’s manual of systematic bacteriology, including Acidobacterium, Geothrix, Holophaga and the recently published *Edaphobacter aggregans*, *Edaphobacter modestus*, *Chloracidobacterium thermophilum* and *Terriglobus roseus* [39]. After the confirmation of the symbiotic microbes in the larva guts, genomic analysis will be possible to reveal the molecular foundations of the relationships between the insect and its microbiome [40]. Furtherly, the roles of the bacterial communities in growth and development, and in the formation of bioactive ingredients of DCXC need further exploration. It is need further for study that endogenetic bacteria play roles in larva pathopoiesis, secondary metabolite formation and fruit body development. Although, pooling the samples from different sampling sites together only was a preliminary approach in study design in the early high-throughput sequencing, which might resulted in the extreme limitations of data, the results of current study also have certain values for the further study. Study design will be improved in the following research of comparing the endogenetic microorganism community in natural DCXC from different areas.

Pharmaceutical substances from natural DCXC are a complex product, just as the DCXC itself is a natural complex of larva and fungal, as well as bacterial communities. Hence, we have good reason to believe that the complicated communities of endogenetic microorganisms in DCXC are related to the secretion of the pharmaceutical substances, and are involved in the growth, development and metabolic process of DCXC. In other words, bioactive ingredients and the infection process of fungus to larva should be related to the endogenetic microorganism community structure of DCXC. In studies of the fungal community structure [15, 41], it was demonstrated that the culture-dependent method only identified limited amounts of microbes [42]. The limitation of the approach resulted few kinds of fungi being found in DCXC. Many studies have indicated a diverse microorganism community in natural DCXC using culture-dependent and independent methods [14, 31]. Obviously, the culture - dependent methods are not sufficient to discover the endogenetic complete community resource, especially the bacterial community. Surprisingly, the DCXC microbiota has so far been poorly explored and exploited for screening bioactive component proposes. In this study, the large proportion of unidentified sequences was found, and the function of these sequences was still unclear. However, illustration of the endogenetic microorganism community structure in DCXC using metagenome data would be beneficial to clarify the relationship between the DCXC and its endogenetic microorganisms, metagenome analysis provides new avenues for the study of the DCXC microorganism community and will reveal the molecular foundations of the relationships between the larva and its microbiome. The unidentified sequences, as a new potential microbial resource, will lay a foundation in the discovery of new drugs and drug leads, and the potential biotechnological application of DCXC microbiota.

## Conclusions

This study revealed an abundant endogenetic fungal and bacterial resources and a variety of genetic information in natural DCXC by high-throughput 454 sequencing technology. In the natural DCXC and its microhabitat, the main bacterial groups were Proteobacteria, Acidobacteria, Bacteroidetes, Actinobacteria and Firmicutes, while the Ascomycota, Basidiomycota and Zygomycota were the main fungal phyla. Proteobacteria presented 68.4, 49.5, 38.9and 35.6 % of all bacteria in the sclerotia, stromata, external mycelial cortices and soil, respectively. And as the main fungi phyla, Ascomycota presented 21.0, 45.6, 26.4and 59.3 % in the sclerotia, stromata, external mycelial cortices and soil, respectively. The bacterial and fungal communities were obviously distinct in each sample and the microorganism communities were more diverse in the environmental sample than in the natural DCXC sample. Microorganisms that had been discovered in natural DCXC will provide sources for screening the new bioactive metabolites and its biotechnological application.

## Methods

### Sample collection and preparation

Natural DCXC in the Nagqu district of Tibet is mainly distributed in six counties: Baqing, Sog, Biru, Jiali, Nagqu and Nie Rong. In particular, with high quality and yield, natural DCXC was distributed in Baqing, Sog, and Biru. The average density of natural DCXC in these counties is 0.42 fruiting body per square meter, with the highest density being four fruiting bodie sper square meter. In current study, about 30 natural DCXC in total were collected with digging them up from the meadow soil of three sampling areas in the Ya-an ethnic township, Baqing County of the South of Nagqu Prefecture of Tibet Autonomous Region (Fig. 1a). In each sampling area, 5–10 natural DCXC were collected from three locations which departed 50–100 m (Fig. 1b). The samples were kept in aseptic valve bags and transported in an ice box to laboratory of Plant Biotechnology R&D Center of Shanghai Jiao Tong University, Shanghai, China. To get rid of the microorganisms inhabiting the surface of natural DCXC, the samples were treated with different disinfectants, including 75 % (*v/v*) ethanol, 2.5 % sodium hypochlorite, 0.01 % mercuric chloride individually, and then washed with sterilized water three times [30]. Subsequently, all the natural DCXC samples collected from three sampling locations were homogenized and divided into stroma, sclerotia and its microhabitat samples, including external mycelial cortices (membrane covering the larvae) and the soil adhering to the surface of the membrane covering DCXC, to investigate the microorganism community (Fig. 1d-i). The average moisture content of the samples were determined by drying to constant weight. The fresh samples were frozen at −20 °C until DNA was extracted. The same sections of natural DCXC samples from 3 different sampling location were pooled together to extract the genomic DNA.

### Genome DNA extraction, PCR amplification and pyrosequencing

Natural DCXC samples were ground with liquid nitrogen to increase the fungus DNA extraction efficiency. About 0.5 g ground samples were used to extract total genomic DNA with a PowerSoil™ soil DNA Isolation Kits (Mo Bio Laboratories, Solana Beach, CA, USA) according to the manufacturer’ s protocol. The V1-V3 region of the 16S rRNA gene was amplified using the primer set 8F (5′-AGAGTTTGATCCTGGCTCAG-3′) and 536R (5′-GWATTACCGCGGCKGCTG-3′) [43] for bacterial community analysis. For fungal community analysis, the ITS sequence, including the partial 18S rRNA gene, was amplified with primer set ITS 1F (5′-CTTGGTCATTTAGAGGAAGTAA-3′) and ITS 4R (5′-TCCTCCGCTTATTGATATGC-3′) [44–46]. For 454-pyrosequencing, a barcode with eight nucleotides was contained in the forward primer, and the requisite adapters A and B were added for the forward and reverse primers, respectively. The PCR amplification was carried out in a total volume of 25 μL PCR mixture containing 12.5 μL Ex Taq DNA polymerase (Takara, Japan), 1 μL bovine serum albumin (25 mg•ml-1), 1 μM of each primer, 1 μL template and 8.5 μL of ultrapure water. The PCR protocol consisted of an initial denaturation step at 95 °C for 5 min; 30 cycles at 94 °C for 30 s and annealing at 55 °C (for the bacterial community study) or 56 °C (for the fungal community study) for 45 s; and extension at 72 °C for 1 min (5 min at 72 °C for the last cycle) [30]. Three replicates of the amplifications were pooled together to minimize the PCR bias. PCR products were detected by electrophoresis through 1.2 % agarose gels and appropriately sized fragments were purified with DNA Gel Extraction Kit (Axygen, Union City, CA, USA). Purified PCR amplicons were sequencing using the Roche-454 GS FLX system, according to the instructions of the Shanghai Personal Biotechnology Co. Ltd.

### Quantitative real-time PCR

Quantitative real-time PCR (qPCR) was used to determine the abundance of bacterial and fungal communities. Universal primer set 338F (5′-ACTCCTACGGGAGGCAGCAG-3′) and 536R [43] were employed to quantify members of total bacteria. Total fungal community abundance was quantified using the same ITS primers used for the pyrosequencing. Standard curves were constructed by serial dilution of linearized plasmids containing the corresponding gene fragment. Gene quantification was performed on a Mx3000P real-time PCR system (Stratagene, La Jolla, CA, USA) with SYBR® Premix ExTaq™ (Takara, Tokyo, Japan). The total reaction volume of 20 μL contained 10 μL SYBR® Premix ExTaq), 0.2 μL bovine serum albumin (25 mg · mL^−1^), 1 μM of each primer, 2 μL template DNA and 6.6 μL of ultrapure water. The thermal programs for assaying total bacterial and fungal community abundance were as described in the pyrosequencing PCR section. The qPCR results were analyzed using MxPro qPCR software version 3.0 (Stratagene, La Jolla, CA, USA). Standard curves had R^2^ >0.99 and the efficiencies for PCR reactions were 95 and 85 % for total bacteria and fungi community, respectively. The copy numbers of the genes were calculated to the unit of “copies per gram dry material”. The Analysis of Variance (ANOVA) and pairwised analysis was conducted with R [47] by commands of “aov” and “pairwise.t.test”, respectively. The *p* values indicated the difference of copy numbers of ITS and 16S rRNA genes were corrected with “holm” [48] method by default of “pairwise.t.test” command and the *p* values < 0.05 were considered significant.

### Analysis of pyrosequencing data

The average length of the raw pyrosequencing data was 550 bp in ITS and 450 bp in 16S rRNA genes sequences. These raw data were processed with the Mothur software package, version 1.27.0 [49]. Briefly, after trimming off the barcode, primers and filtering out the sequences with a quality score of any base below 25 with the “trim.seqs” command. The sequences were also trimmed if the fragment length beyond of 450 bp to 600 bp and 400 bp to 550 bp for ITS sequence and 16S rRNA genes, respectively. After potential chimeric sequences were checked with the chimera.slayer command, 44,658 16S rRNA genes and 51,584 ITS high quality sequences were obtained. The pyrosequencing reads of 16S rRNA gene were aligned against SILVA [50] with the command of “align.seqs”. After the distance matrix was calculated with the “dist.seqs” command, the qualified sequences were clustered into OTUs at 97, 95 and 90 % similarity with the “cluster” command, respectively. The OTUs of ITS sequences were picked with USEARCH61 [51] without alignment. After the bacterial 16S rRNA gene and ITS sequences in each sample were normalized to the minimum of 9042 and 8585, respectively, diversity indices (Shannon-Weiner), richness estimator (Chao1) and the evenness index (ACE) were calculated using the “summary.seqs” command for each sample. Taxonomy information was assigned to 16S rRNA gene and ITS sequences using localblast program against SILVA [50] and UNITE [52] databases, respectively with the “classify.seqs” command. Finally, weighted-Principal coordinates analysis was performed using QIIME version 1.7 [53].

## Additional files

Additional file 1: Figure S1. Rarefaction curves of 16S rRNA genes and ITS sequences. (a), (c) and (e) are the rarefaction curves of the 16S rRNA genes at cut off values of 97, 95 and 95 % similarity. While (b), (d) and (f) are the rarefaction curves for ITS sequences at cut off values of 97, 95 and 95 % similarity. In each panel, the x-axis represents the number of sequences and the y-axis represents the number of operational taxonomic units (OTUs) determined at a particular cut off value. (TIF 396 kb) Additional file 2: Figure S2. Operational taxonomic units (OTUs) rank curves of 16S rRNA gene (a) and ITS sequences (b) at a 97 % cut off value. In each figure, the x-axis represents the OTUs listed as the abundance in descending order and the y-axis represents the relative abundance of each OTU. The OTU rank curve could explain the diversity and the evenness of the organism community. The width of the curve reveals the diversity of the organism community along the x-axis; a wider curve indicates more diversity. Evenness of the organism community is suggested by the pattern of the curve. A flatter curve suggests a more even organism community. (TIF 216 kb) Additional file 3: Table S1. The classification of the bacteria detected in each sample of natural DCXC at the genus level. CT, larva; ZZ, stroma; JP, the membrane of DCXC; soil, bacteria isolated from the surface of natural DCXC. The percentages following the genus names are the proportions of each microbe group in the total obtained sequences of each sample. (DOCX 44 kb) Additional file 4: Table S2. The classification of the fungi detected in each sample of natural DCXC at the genus level. CT, larva; ZZ, stroma; JP, the membrane of DCXC; soil, bacteria isolated from the surface of natural DCXC. The percentages following the genus names are the proportion of each microbe group in the total obtained sequences of each sample. (DOCX 31 kb) Additional file 5: Figure S3. Unweighted Pair Group Method with Arithmetic Averages (UPGMA) dendrogram constructed from ThetaYC distances of bacterial communities in each sample (A) and fungal community (B). The values were shown above the lines indicate differences between the organism community structure in each sample. (TIF 106 kb) Additional file 6: Figure S4. Venn diagram of the shared operational taxonomic units (OTUs) of the bacterial (a) and fungal (b) community in each sample from DCXC. The OTUs were generated with a 97 % similarity cut off value. Different colors show the different samples and the numbers in the across area are the number of OTUs shared by different samples. (TIF 434 kb)

## Abbreviations

DCXC: Natural *Ophiocordyceps sinensis* (syn. *Cordyceps sinensis*)
ITS: Internal transcribed spacer
TCMM: Traditional Chinese medicinal materials
qPCR: Quantitative real-time PCR
OTU: Operational taxonomic units
PCoA: Principal coordinates analysis
UPGMA: Unweighted pair group method with arithmetic averages
PCR: Polymerase chain reaction
