# Supplementary figures and images for: Pyrosequencing analysis revealed complex endogenetic microorganism community from natural DongChong XiaCao and its microhabitat
Source: BMC Microbiol. 2016 Aug 26;16(1):196. doi: 10.1186/s12866-016-0813-5 (PMC5002179; doi:10.1186/s12866-016-0813-5)

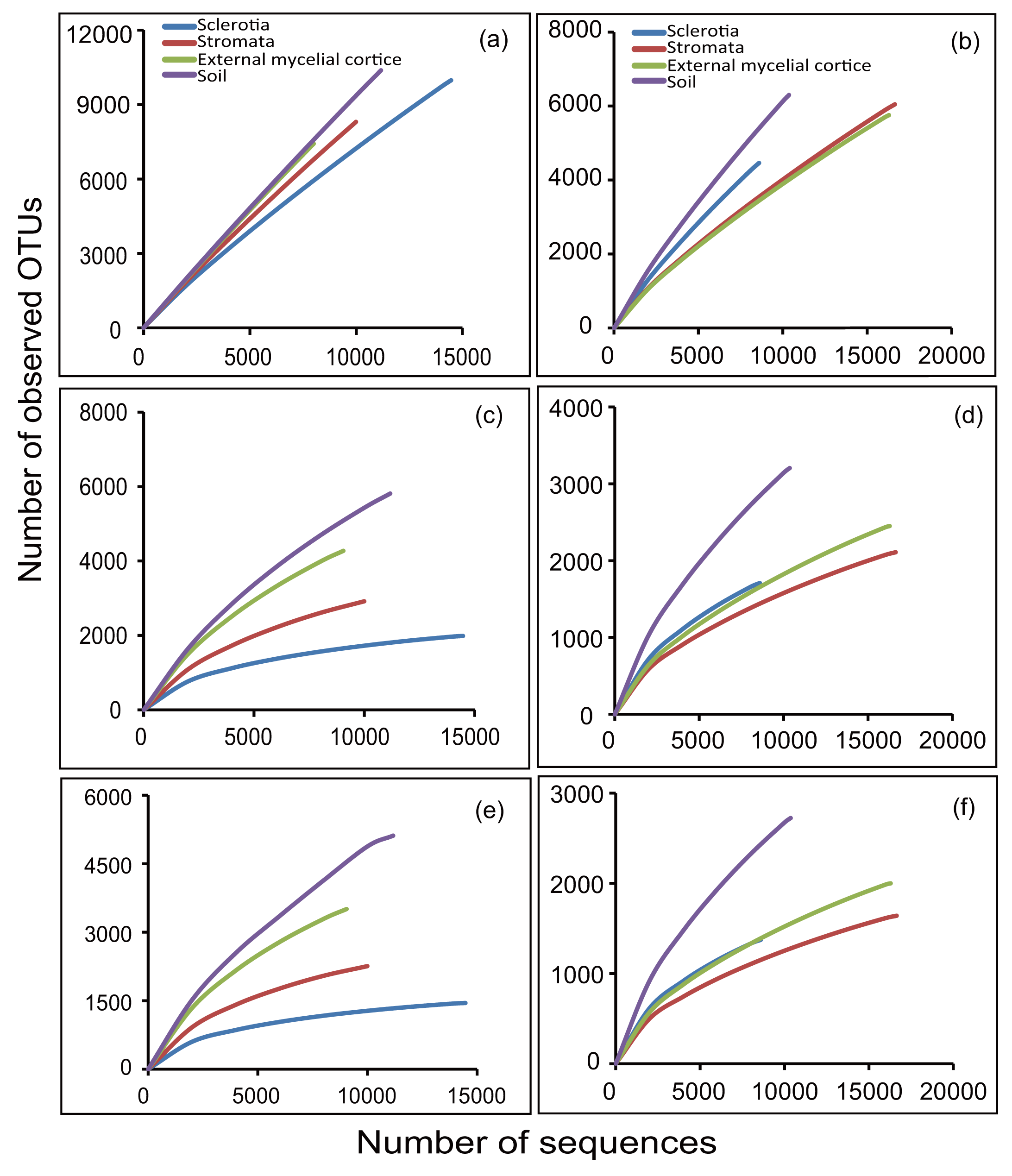

Supplement: Additional file 1: Figure S1. — Rarefaction curves of 16S rRNA genes and ITS sequences. (a), (c) and (e) are the rarefaction curves of the 16S rRNA genes at cut off values of 97, 95 and 95 % similarity. While (b), (d) and (f) are the rarefaction curves for ITS sequences at cut off values of 97, 95 and 95 % similarity. In each panel, the x-axis represents the number of sequences and the y-axis represents the number of operational taxonomic units (OTUs) determined at a particular cut off value. (TIF 396 kb) [file 12866_2016_813_MOESM1_ESM.tif]

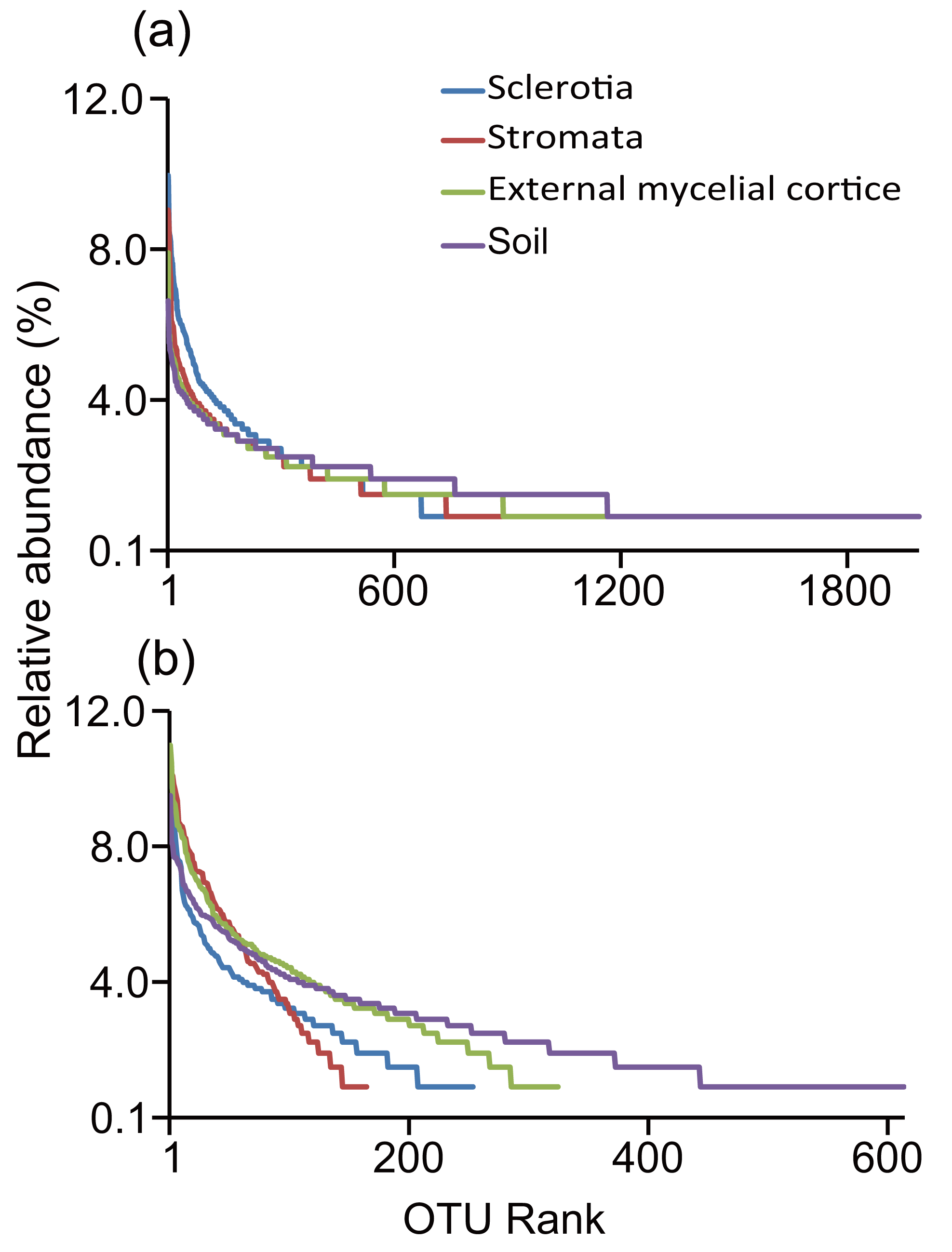

Supplement: Additional file 2: Figure S2. — Operational taxonomic units (OTUs) rank curves of 16S rRNA gene (a) and ITS sequences (b) at a 97 % cut off value. In each figure, the x-axis represents the OTUs listed as the abundance in descending order and the y-axis represents the relative abundance of each OTU. The OTU rank curve could explain the diversity and the evenness of the organism community. The width of the curve reveals the diversity of the organism community along the x-axis; a wider curve indicates more diversity. Evenness of the organism community is suggested by the pattern of the curve. A flatter curve suggests a more even organism community. (TIF 216 kb) [file 12866_2016_813_MOESM2_ESM.tif]

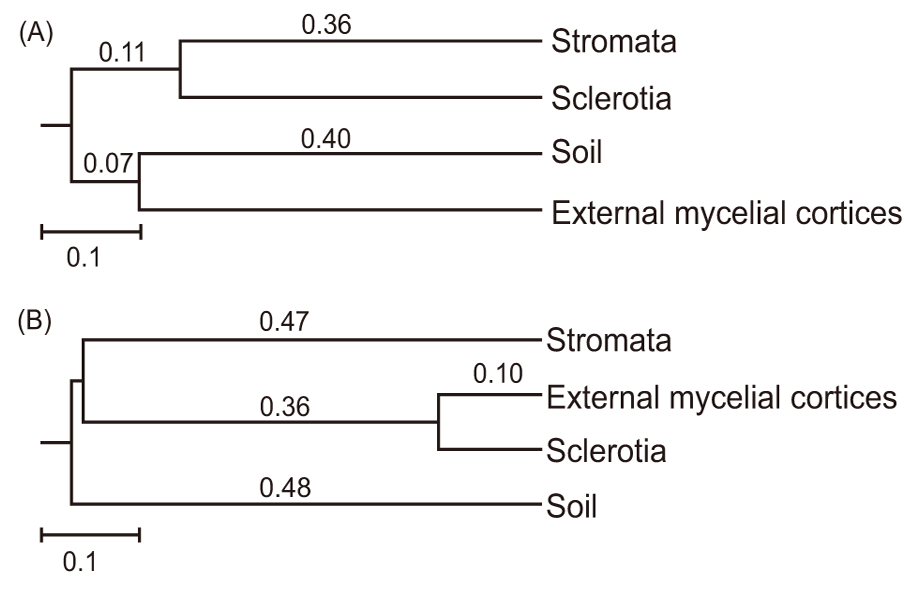

Supplement: Additional file 5: Figure S3. — Unweighted Pair Group Method with Arithmetic Averages (UPGMA) dendrogram constructed from ThetaYC distances of bacterial communities in each sample (A) and fungal community (B). The values were shown above the lines indicate differences between the organism community structure in each sample. (TIF 106 kb) [file 12866_2016_813_MOESM5_ESM.tif]

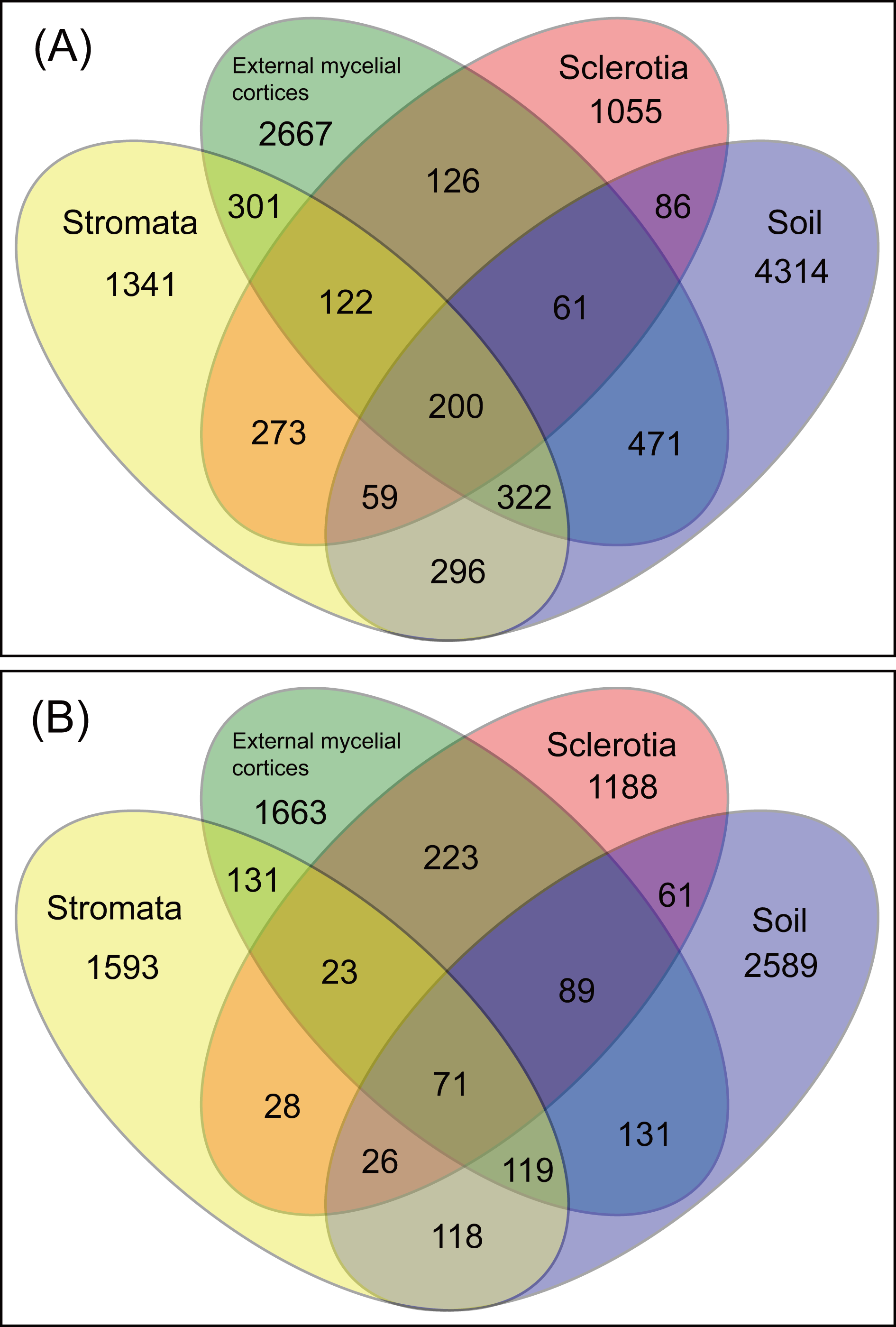

Supplement: Additional file 6: Figure S4. — Venn diagram of the shared operational taxonomic units (OTUs) of the bacterial (a) and fungal (b) community in each sample from DCXC. The OTUs were generated with a 97 % similarity cut off value. Different colors show the different samples and the numbers in the across area are the number of OTUs shared by different samples. (TIF 434 kb) [file 12866_2016_813_MOESM6_ESM.tif]
