# Supplementary material for: Pyrosequencing analysis revealed complex endogenetic microorganism community from natural DongChong XiaCao and its microhabitat
Source: BMC Microbiol. 2016 Aug 26;16(1):196. doi: 10.1186/s12866-016-0813-5 (PMC5002179; doi:10.1186/s12866-016-0813-5)
Supplement: Additional file 3: Table S1. — The classification of the bacteria detected in each sample of natural DCXC at the genus level. CT, larva; ZZ, stroma; JP, the membrane of DCXC; soil, bacteria isolated from the surface of natural DCXC. The percentages following the genus names are the proportions of each microbe group in the total obtained sequences of each sample. (DOCX 44 kb) [file 12866_2016_813_MOESM3_ESM.docx]

Table S1 The classification of the bacteria detected in each sample of the *O. sinensis* at the genus level. CT-larva; ZZ-stroma; JP-membrane of the DCXC; soil-isolated from the surface of *O. sinensis*. The percentage following the genus names were the proportion of each microbe group in the total obtained sequences of each sample.

| **CT** | |  | **ZZ** | |  | **JP** | |  | **Soil** | |
| --- | --- | --- | --- | --- | --- | --- | --- | --- | --- | --- |
| **Genus** | **Percentage** | | **Genus** | **Percentage** |  | **Genus** | **Percentage** | | **Genus** | **Percentage** |
| Other | 29.06% |  | Other | 54.63% |  | Other | 78.54% |  | Other | 80.73% |
| Pseudomonas | 9.24% |  | Pedobacter | 8.05% |  | Thermomonas | 3.43% |  | Ferruginibacter | 3.07% |
| Rhodoferax | 9.11% |  | Rhodoferax | 6.91% |  | Gemmatimonas | 2.13% |  | Gemmatimonas | 2.22% |
| Pedobacter | 3.42% |  | Variovorax | 4.41% |  | Ferruginibacter | 1.64% |  | Terrimonas | 1.93% |
| Collimonas | 2.78% |  | Mucilaginibacter | 3.96% |  | Terrimonas | 1.39% |  | Methylibium | 1.13% |
| Janthinobacterium | 2.37% |  | Chryseobacterium | 2.02% |  | Opitutus | 1.07% |  | Opitutus | 0.88% |
| Herbaspirillum | 2.30% |  | Ferruginibacter | 1.23% |  | Methylibium | 0.98% |  | Flavobacterium | 0.70% |
| Sphingomonas | 2.29% |  | Methylibium | 0.98% |  | Nitrospira | 0.71% |  | Steroidobacter | 0.45% |
| Variovorax | 2.28% |  | Gemmatimonas | 0.97% |  | Rhodoferax | 0.62% |  | Nitrospira | 0.42% |
| Streptococcus | 2.21% |  | Pelomonas | 0.95% |  | Massilia | 0.60% |  | Zavarzinella | 0.40% |
| Polaromonas | 1.76% |  | Terrimonas | 0.91% |  | Sphingomonas | 0.48% |  | Sphingomonas | 0.37% |
| Alkanindiges | 1.72% |  | Sphingobacterium | 0.90% |  | Zavarzinella | 0.46% |  | Massilia | 0.31% |
| Acidovorax | 1.55% |  | Brevundimonas | 0.86% |  | Caulobacter | 0.45% |  | Variovorax | 0.30% |
| Caulobacter | 1.49% |  | Flavobacterium | 0.73% |  | Rudaea | 0.45% |  | Gemmata | 0.26% |
| Staphylococcus | 1.42% |  | Herbaspirillum | 0.70% |  | Flavobacterium | 0.43% |  | Caulobacter | 0.25% |
| Sphingobacterium | 1.38% |  | Rudaea | 0.69% |  | Variovorax | 0.41% |  | Byssovorax | 0.25% |
| Brevundimonas | 1.33% |  | Duganella | 0.61% |  | Pedobacter | 0.32% |  | Mucilaginibacter | 0.25% |
| Flavobacterium | 1.23% |  | Mesorhizobium | 0.53% |  | Curvibacter | 0.30% |  | Pirellula | 0.25% |
| Lactobacillus | 1.19% |  | Chitinophaga | 0.49% |  | Haliscomenobacter | 0.28% |  | Haliscomenobacter | 0.24% |
| Mesorhizobium | 1.16% |  | Polaromonas | 0.48% |  | Phenylobacterium | 0.27% |  | Rudaea | 0.23% |
| Enterobacter | 0.97% |  | Caulobacter | 0.42% |  | Asticcacaulis | 0.24% |  | Spirosoma | 0.20% |
| Thermomonas | 0.89% |  | Opitutus | 0.41% |  | Duganella | 0.23% |  | Curvibacter | 0.19% |
| Mucilaginibacter | 0.85% |  | Nitrospira | 0.32% |  | Pseudomonas | 0.21% |  | Dokdonella | 0.19% |
| Ochrobactrum | 0.85% |  | Massilia | 0.31% |  | Gemmata | 0.20% |  | Mycobacterium | 0.19% |
| Methylibium | 0.75% |  | Spirosoma | 0.31% |  | Steroidobacter | 0.17% |  | Pseudonocardia | 0.19% |
| Rhizobium | 0.72% |  | Acidovorax | 0.29% |  | Polaromonas | 0.17% |  | Iamia | 0.18% |
| Mycobacterium | 0.55% |  | Asticcacaulis | 0.28% |  | Pirellula | 0.16% |  | Brevundimonas | 0.17% |
| Serratia | 0.54% |  | Dyadobacter | 0.28% |  | Kofleria | 0.16% |  | Duganella | 0.17% |
| Neisseria | 0.53% |  | Rhizobium | 0.28% |  | Mesorhizobium | 0.15% |  | Rhodoferax | 0.16% |
| Corynebacterium | 0.53% |  | Devosia | 0.25% |  | Marmoricola | 0.14% |  | Mesorhizobium | 0.14% |
| Duganella | 0.51% |  | Ochrobactrum | 0.25% |  | Byssovorax | 0.13% |  | Kineosporia | 0.14% |
| Propionibacterium | 0.50% |  | Sphingomonas | 0.23% |  | Schlesneria | 0.13% |  | Agromonas | 0.13% |
| Clostridium | 0.45% |  | Phenylobacterium | 0.21% |  | Herbaspirillum | 0.13% |  | Phenylobacterium | 0.13% |
| Ottowia | 0.45% |  | Haliscomenobacter | 0.17% |  | Mucilaginibacter | 0.12% |  | Thermomonas | 0.13% |
| Chryseobacterium | 0.43% |  | Pseudonocardia | 0.17% |  | Thiobacter | 0.12% |  | Humicoccus | 0.12% |
| Rudaea | 0.39% |  | Pirellula | 0.16% |  | Singulisphaera | 0.12% |  | Pseudoxanthomonas | 0.11% |
| Bacillus | 0.35% |  | Dokdonella | 0.16% |  | Janthinobacterium | 0.11% |  | Schlesneria | 0.11% |
| Enterococcus | 0.35% |  | Thermomonas | 0.16% |  | Dokdonella | 0.10% |  | Pedobacter | 0.10% |
| Stenotrophomonas | 0.31% |  | Undibacterium | 0.16% |  | Pseudonocardia | 0.10% |  | Sorangium | 0.10% |
| Dokdonella | 0.31% |  | Humicoccus | 0.15% |  | Leifsonia | 0.10% |  | Flavisolibacter | 0.09% |
| Ferruginibacter | 0.28% |  | Janthinobacterium | 0.15% |  | Kineosporia | 0.08% |  | Friedmanniella | 0.09% |
| Mogibacterium | 0.28% |  | Sphingopyxis | 0.14% |  | Luteolibacter | 0.08% |  | Kofleria | 0.09% |
| Terrimonas | 0.27% |  | Pseudochrobactrum | 0.14% |  | Iamia | 0.07% |  | Asticcacaulis | 0.08% |
| Pseudochrobactrum | 0.27% |  | Labrys | 0.13% |  | Pedomicrobium | 0.07% |  | Marmoricola | 0.08% |
| Microbacterium | 0.25% |  | Gemmata | 0.12% |  | Pelomonas | 0.07% |  | Pedomicrobium | 0.08% |
| Subdoligranulum | 0.25% |  | Singulisphaera | 0.12% |  | Serratia | 0.07% |  | Caenimonas | 0.08% |
| Fusobacterium | 0.23% |  | Curvibacter | 0.11% |  | Aquicella | 0.07% |  | Luteibacter | 0.07% |
| Bosea | 0.21% |  | Mycobacterium | 0.11% |  | Dactylosporangium | 0.07% |  | Methylobacterium | 0.07% |
| Lactococcus | 0.21% |  | Pseudomonas | 0.11% |  | Caenimonas | 0.06% |  | Phyllobacterium | 0.07% |
| Sphingopyxis | 0.21% |  | Aminobacter | 0.11% |  | Nannocystis | 0.06% |  | Azospira | 0.06% |
| Massilia | 0.21% |  | Hyphomicrobium | 0.10% |  | Nocardioides | 0.06% |  | Caldilinea | 0.06% |
| Gemmatimonas | 0.20% |  | Microbacterium | 0.10% |  | Filimonas | 0.05% |  | Haliangium | 0.06% |
| Pelomonas | 0.19% |  | Ralstonia | 0.10% |  | Conexibacter | 0.05% |  | Hyphomicrobium | 0.06% |
| Opitutus | 0.18% |  | Phyllobacterium | 0.09% |  | Dyadobacter | 0.05% |  | Nannocystis | 0.06% |
| Leifsonia | 0.18% |  | Nocardioides | 0.09% |  | Devosia | 0.05% |  | Nocardioides | 0.06% |
| Hyphomicrobium | 0.17% |  | Conexibacter | 0.08% |  | Mycobacterium | 0.04% |  | Pseudomonas | 0.06% |
| Blautia | 0.17% |  | Stenotrophomonas | 0.08% |  | Humicoccus | 0.04% |  | Ideonella | 0.05% |
| Turicibacter | 0.17% |  | Bosea | 0.08% |  | Haliangium | 0.04% |  | Pelomonas | 0.05% |
| Chitinophaga | 0.16% |  | Steroidobacter | 0.07% |  | Bdellovibrio | 0.04% |  | Solirubrobacter | 0.05% |
| Nitrospira | 0.15% |  | Iamia | 0.07% |  | Novosphingobium | 0.04% |  | Staphylococcus | 0.05% |
| Cardiobacterium | 0.15% |  | Pseudoxanthomonas | 0.07% |  | Labrys | 0.04% |  | Filimonas | 0.04% |
| Luteolibacter | 0.15% |  | Schlesneria | 0.07% |  | Undibacterium | 0.04% |  | Sandarakinorhabdus | 0.04% |
| Oscillibacter | 0.15% |  | Fluviicola | 0.07% |  | Bacillus | 0.04% |  | Aquabacterium | 0.03% |
| Capnocytophaga | 0.14% |  | Burkholderia | 0.07% |  | Hyalangium | 0.04% |  | Bacteriovorax | 0.03% |
| Kingella | 0.14% |  | Rhodobacter | 0.07% |  | Caldilinea | 0.03% |  | Bdellovibrio | 0.03% |
| Methylotenera | 0.14% |  | Zavarzinella | 0.06% |  | Hyphomicrobium | 0.03% |  | Fluviicola | 0.03% |
| Phenylobacterium | 0.13% |  | Aeromicrobium | 0.06% |  | Aeromicrobium | 0.03% |  | Janthinobacterium | 0.03% |
| Sphingobium | 0.13% |  | Aquicella | 0.06% |  | Sporichthya | 0.03% |  | Rhodopila | 0.03% |
| Sporacetigenium | 0.13% |  | Marmoricola | 0.05% |  | Herminiimonas | 0.03% |  | Actinoplanes | 0.03% |
| Burkholderia | 0.13% |  | Caldilinea | 0.05% |  | Cellvibrio | 0.03% |  | Aeromicrobium | 0.03% |
| Allobaculum | 0.13% |  | Solirubrobacter | 0.05% |  | Hymenobacter | 0.03% |  | Blastochloris | 0.03% |
| Brevibacillus | 0.13% |  | Filimonas | 0.05% |  | Brevundimonas | 0.02% |  | Geothrix | 0.03% |
| Schlesneria | 0.11% |  | Thiobacter | 0.05% |  | Pseudoxanthomonas | 0.02% |  | Knoellia | 0.03% |
| Paenibacillus | 0.11% |  | Bacillus | 0.05% |  | Aquabacterium | 0.02% |  | Methylocapsa | 0.03% |
| Aminobacter | 0.10% |  | Luteolibacter | 0.05% |  | Sphingobium | 0.02% |  | Microlunatus | 0.03% |
| Methylobacterium | 0.09% |  | Lysobacter | 0.05% |  | Chitinimonas | 0.02% |  | Novosphingobium | 0.03% |
| Solirubrobacter | 0.09% |  | Kineosporia | 0.04% |  | Chitinophaga | 0.02% |  | Patulibacter | 0.03% |
| Devosia | 0.09% |  | Bacteriovorax | 0.04% |  | Anaeromyxobacter | 0.02% |  | Sphingobium | 0.03% |
| Aggregatibacter | 0.09% |  | Novosphingobium | 0.04% |  | Corynebacterium | 0.02% |  | Streptomyces | 0.03% |
| Prevotella | 0.09% |  | Afipia | 0.04% |  | Inquilinus | 0.02% |  | Thiobacter | 0.03% |
| Ralstonia | 0.09% |  | Collimonas | 0.04% |  | Spirosoma | 0.01% |  | Verrucomicrobium | 0.03% |
| Asticcacaulis | 0.08% |  | Delftia | 0.04% |  | Sorangium | 0.01% |  | Afipia | 0.02% |
| Delftia | 0.08% |  | Paucibacter | 0.04% |  | Flavisolibacter | 0.01% |  | Amaricoccus | 0.02% |
| Haloferula | 0.08% |  | Byssovorax | 0.03% |  | Phyllobacterium | 0.01% |  | Bradyrhizobium | 0.02% |
| Curvibacter | 0.07% |  | Sorangium | 0.03% |  | Geothrix | 0.01% |  | Burkholderia | 0.02% |
| Dyadobacter | 0.07% |  | Kofleria | 0.03% |  | Verrucomicrobium | 0.01% |  | Chitinimonas | 0.02% |
| Legionella | 0.07% |  | Pedomicrobium | 0.03% |  | Afipia | 0.01% |  | Chitinophaga | 0.02% |
| Planomicrobium | 0.07% |  | Aquabacterium | 0.03% |  | Collimonas | 0.01% |  | Chryseobacterium | 0.02% |
| Thiobacter | 0.06% |  | Aerococcus | 0.03% |  | Ochrobactrum | 0.01% |  | Conexibacter | 0.02% |
| Pseudoxanthomonas | 0.05% |  | Agrococcus | 0.03% |  | Acidovorax | 0.01% |  | Dyadobacter | 0.02% |
| Aquabacterium | 0.05% |  | Enterobacter | 0.03% |  | Alkanindiges | 0.01% |  | Ferribacterium | 0.02% |
| Anaerovorax | 0.05% |  | Caenimonas | 0.02% |  | Arthrobacter | 0.01% |  | Labrys | 0.02% |
| Elizabethkingia | 0.05% |  | Luteibacter | 0.02% |  | Aspromonas | 0.01% |  | Niastella | 0.02% |
| Eubacterium | 0.05% |  | Methylobacterium | 0.02% |  | Catelliglobosispora | 0.01% |  | Paracraurococcus | 0.02% |
| Kocuria | 0.05% |  | Staphylococcus | 0.02% |  | Clostridium | 0.01% |  | Phycicoccus | 0.02% |
| Leuconostoc | 0.05% |  | Bdellovibrio | 0.02% |  | Enterobacter | 0.01% |  | Prosthecobacter | 0.02% |
| Phyllobacterium | 0.05% |  | Sphingobium | 0.02% |  | Kaistia | 0.01% |  | Rhizobacter | 0.02% |
| Undibacterium | 0.05% |  | Chitinimonas | 0.02% |  | Lentzea | 0.01% |  | Rhizobium | 0.02% |
| Janibacter | 0.05% |  | Sporotalea | 0.02% |  | Lysobacter | 0.01% |  | Sporichthya | 0.02% |
| Rhodobacter | 0.05% |  | Citricoccus | 0.02% |  | Paenibacillus | 0.01% |  | Sporotalea | 0.02% |
| Zavarzinella | 0.04% |  | Clostridium | 0.02% |  | Paucibacter | 0.01% |  | Undibacterium | 0.02% |
| Actinomyces | 0.04% |  | Dyella | 0.02% |  | Planctomyces | 0.01% |  | Anaeromyxobacter | 0.01% |
| Aerococcus | 0.04% |  | Mitsuaria | 0.02% |  | Propionibacterium | 0.01% |  | Asanoa | 0.01% |
| Aquicella | 0.04% |  | Streptococcus | 0.02% |  | Ramlibacter | 0.01% |  | Bacillus | 0.01% |
| Atopostipes | 0.04% |  | Friedmanniella | 0.01% |  | Rhodanobacter | 0.01% |  | Blastococcus | 0.01% |
| Collinsella | 0.04% |  | Ideonella | 0.01% |  | Rhodopirellula | 0.01% |  | Collimonas | 0.01% |
| Kaistia | 0.04% |  | Actinoplanes | 0.01% |  | Roseomonas | 0.01% |  | Devosia | 0.01% |
| Leptotrichia | 0.04% |  | Patulibacter | 0.01% |  | \ | \ |  | Herbaspirillum | 0.01% |
| Paucibacter | 0.04% |  | Anaeromyxobacter | 0.01% |  | \ | \ |  | Herminiimonas | 0.01% |
| Spirosoma | 0.03% |  | Alkanindiges | 0.01% |  | \ | \ |  | Hyalangium | 0.01% |
| Novosphingobium | 0.03% |  | Amycolatopsis | 0.01% |  | \ | \ |  | Kribbella | 0.01% |
| Acinetobacter | 0.03% |  | Aurantimonas | 0.01% |  | \ | \ |  | Luteolibacter | 0.01% |
| Luteimonas | 0.03% |  | Brachybacterium | 0.01% |  | \ | \ |  | Methylocystis | 0.01% |
| Micrococcus | 0.03% |  | Defluviicoccus | 0.01% |  | \ | \ |  | Niabella | 0.01% |
| Olsenella | 0.03% |  | Deinococcus | 0.01% |  | \ | \ |  | Ochrobactrum | 0.01% |
| Singulisphaera | 0.03% |  | Emticicia | 0.01% |  | \ | \ |  | Porphyromonas | 0.01% |
| Arthrobacter | 0.03% |  | Hymenobacter | 0.01% |  | \ | \ |  | Serratia | 0.01% |
| Brevibacterium | 0.03% |  | Janibacter | 0.01% |  | \ | \ |  | Singulisphaera | 0.01% |
| Cellvibrio | 0.03% |  | Ktedonobacter | 0.01% |  | \ | \ |  | Stenotrophomonas | 0.01% |
| Geobacter | 0.03% |  | Lactococcus | 0.01% |  | \ | \ |  | \ | \ |
| Inquilinus | 0.03% |  | Legionella | 0.01% |  | \ | \ |  | \ | \ |
| Rothia | 0.03% |  | Leifsonia | 0.01% |  | \ | \ |  | \ | \ |
| Steroidobacter | 0.02% |  | Lysinibacillus | 0.01% |  | \ | \ |  | \ | \ |
| Pseudonocardia | 0.02% |  | Paenibacillus | 0.01% |  | \ | \ |  | \ | \ |
| Flavisolibacter | 0.02% |  | Parapedobacter | 0.01% |  | \ | \ |  | \ | \ |
| Kofleria | 0.02% |  | Planctomyces | 0.01% |  | \ | \ |  | \ | \ |
| Nocardioides | 0.02% |  | Propionibacterium | 0.01% |  | \ | \ |  | \ | \ |
| Patulibacter | 0.02% |  | Ramlibacter | 0.01% |  | \ | \ |  | \ | \ |
| Amaricoccus | 0.02% |  | Rhodanobacter | 0.01% |  | \ | \ |  | \ | \ |
| Asteroleplasma | 0.02% |  | Rhodococcus | 0.01% |  | \ | \ |  | \ | \ |
| Brachybacterium | 0.02% |  | Sediminibacterium | 0.01% |  | \ | \ |  | \ | \ |
| Catenibacterium | 0.02% |  | Shinella | 0.01% |  | \ | \ |  | \ | \ |
| Cupriavidus | 0.02% |  | Sinorhizobium | 0.01% |  | \ | \ |  | \ | \ |
| Deinococcus | 0.02% |  | Thermoleophilum | 0.01% |  | \ | \ |  | \ | \ |
| Demequina | 0.02% |  | \ | \ |  | \ | \ |  | \ | \ |
| Derxia | 0.02% |  | \ | \ |  | \ | \ |  | \ | \ |
| Dietzia | 0.02% |  | \ | \ |  | \ | \ |  | \ | \ |
| Enhydrobacter | 0.02% |  | \ | \ |  | \ | \ |  | \ | \ |
| Escherichia/Shigella | 0.02% |  | \ | \ |  | \ | \ |  | \ | \ |
| Petrobacter | 0.02% |  | \ | \ |  | \ | \ |  | \ | \ |
| Shinella | 0.02% |  | \ | \ |  | \ | \ |  | \ | \ |
| Stackebrandtia | 0.02% |  | \ | \ |  | \ | \ |  | \ | \ |
| Turneriella | 0.02% |  | \ | \ |  | \ | \ |  | \ | \ |
| Gemmata | 0.01% |  | \ | \ |  | \ | \ |  | \ | \ |
| Nannocystis | 0.01% |  | \ | \ |  | \ | \ |  | \ | \ |
| Filimonas | 0.01% |  | \ | \ |  | \ | \ |  | \ | \ |
| Bradyrhizobium | 0.01% |  | \ | \ |  | \ | \ |  | \ | \ |
| Anaeromyxobacter | 0.01% |  | \ | \ |  | \ | \ |  | \ | \ |
| Hyalangium | 0.01% |  | \ | \ |  | \ | \ |  | \ | \ |
| Azospirillum | 0.01% |  | \ | \ |  | \ | \ |  | \ | \ |
| Dialister | 0.01% |  | \ | \ |  | \ | \ |  | \ | \ |
| Faecalibacterium | 0.01% |  | \ | \ |  | \ | \ |  | \ | \ |
| Leucobacter | 0.01% |  | \ | \ |  | \ | \ |  | \ | \ |
| Ornithinimicrobium | 0.01% |  | \ | \ |  | \ | \ |  | \ | \ |
| Porphyrobacter | 0.01% |  | \ | \ |  | \ | \ |  | \ | \ |
| Saccharopolyspora | 0.01% |  | \ | \ |  | \ | \ |  | \ | \ |
| Sediminibacterium | 0.01% |  | \ | \ |  | \ | \ |  | \ | \ |
| Marmoricola | 0.01% |  | \ | \ |  | \ | \ |  | \ | \ |
| Caenimonas | 0.01% |  | \ | \ |  | \ | \ |  | \ | \ |
| Bacteriovorax | 0.01% |  | \ | \ |  | \ | \ |  | \ | \ |
| Fluviicola | 0.01% |  | \ | \ |  | \ | \ |  | \ | \ |
| Verrucomicrobium | 0.01% |  | \ | \ |  | \ | \ |  | \ | \ |
| Labrys | 0.01% |  | \ | \ |  | \ | \ |  | \ | \ |
| Sporichthya | 0.01% |  | \ | \ |  | \ | \ |  | \ | \ |
| Abiotrophia | 0.01% |  | \ | \ |  | \ | \ |  | \ | \ |
| Achromobacter | 0.01% |  | \ | \ |  | \ | \ |  | \ | \ |
| Anaerotruncus | 0.01% |  | \ | \ |  | \ | \ |  | \ | \ |
| Anoxybacillus | 0.01% |  | \ | \ |  | \ | \ |  | \ | \ |
| Arsenicicoccus | 0.01% |  | \ | \ |  | \ | \ |  | \ | \ |
| Comamonas | 0.01% |  | \ | \ |  | \ | \ |  | \ | \ |
| Defluvibacter | 0.01% |  | \ | \ |  | \ | \ |  | \ | \ |
| Defluviicoccus | 0.01% |  | \ | \ |  | \ | \ |  | \ | \ |
| Granulicatella | 0.01% |  | \ | \ |  | \ | \ |  | \ | \ |
| Leptospira | 0.01% |  | \ | \ |  | \ | \ |  | \ | \ |
| Lysinibacillus | 0.01% |  | \ | \ |  | \ | \ |  | \ | \ |
| Mitsuaria | 0.01% |  | \ | \ |  | \ | \ |  | \ | \ |
| Nevskia | 0.01% |  | \ | \ |  | \ | \ |  | \ | \ |
| Nitrobacter | 0.01% |  | \ | \ |  | \ | \ |  | \ | \ |
| Parvibaculum | 0.01% |  | \ | \ |  | \ | \ |  | \ | \ |
| Pediococcus | 0.01% |  | \ | \ |  | \ | \ |  | \ | \ |
| Pseudoclavibacter | 0.01% |  | \ | \ |  | \ | \ |  | \ | \ |
| Rhodococcus | 0.01% |  | \ | \ |  | \ | \ |  | \ | \ |
| Roseomonas | 0.01% |  | \ | \ |  | \ | \ |  | \ | \ |
| Selenomonas | 0.01% |  | \ | \ |  | \ | \ |  | \ | \ |
| Sharpea | 0.01% |  | \ | \ |  | \ | \ |  | \ | \ |
| Skermanella | 0.01% |  | \ | \ |  | \ | \ |  | \ | \ |
