# Supplementary material for: Pyrosequencing analysis revealed complex endogenetic microorganism community from natural DongChong XiaCao and its microhabitat
Source: BMC Microbiol. 2016 Aug 26;16(1):196. doi: 10.1186/s12866-016-0813-5 (PMC5002179; doi:10.1186/s12866-016-0813-5)
Supplement: Additional file 4: Table S2. — The classification of the fungi detected in each sample of natural DCXC at the genus level. CT, larva; ZZ, stroma; JP, the membrane of DCXC; soil, bacteria isolated from the surface of natural DCXC. The percentages following the genus names are the proportion of each microbe group in the total obtained sequences of each sample. (DOCX 31 kb) [file 12866_2016_813_MOESM4_ESM.docx]

Table S2 The classification of the fungi detected in each sample of the *O. sinensis* at the genus level. CT-larva; ZZ-stroma; JP-membrane of the DCXC; soil-isolated from the surface of *O. sinensis*. The percentage following the genus names were the proportion of each microbe group in the total obtained sequences of each sample.

| **CT** | |  | **ZZ** | |  | **JP** | |  | **Soil** | |
| --- | --- | --- | --- | --- | --- | --- | --- | --- | --- | --- |
| **Genus** | **Percentage** |  | **Genus** | **Percentage** |  | **Genus** | **Percentage** |  | **Genus** | **Percentage** |
| Unknown | 73.64% |  | Unknown | 85.52% |  | Unknown | 87.55% |  | Unknown | 67.32% |
| No blast hit | 19.09% |  | No blast hit | 1.64% |  | No blast hit | 2.05% |  | No blast hit | 2.82% |
| Entoloma | 2.62% |  | Exophiala | 2.90% |  | Entoloma | 3.47% |  | Cladophialophora | 3.30% |
| Cladophialophora | 0.85% |  | Cladophialophora | 1.77% |  | Tetracladium | 1.38% |  | Verrucaria | 3.10% |
| Tetracladium | 0.70% |  | Phaeomollisia | 1.45% |  | Cladophialophora | 0.90% |  | Sebacina | 2.96% |
| Capronia | 0.42% |  | Tetracladium | 0.85% |  | Exophiala | 0.67% |  | Gyoerffyella | 2.33% |
| Hypoderma | 0.22% |  | Archaeorhizomyces | 0.79% |  | Hypoderma | 0.64% |  | Exophiala | 1.56% |
| Pseudogymnoascus | 0.19% |  | Trichosporon | 0.79% |  | Ramariopsis | 0.39% |  | Capronia | 1.51% |
| Russula | 0.19% |  | Collembolispora | 0.70% |  | Phaeomollisia | 0.26% |  | Xylogone | 1.22% |
| Malassezia | 0.17% |  | Entrophospora | 0.65% |  | Cryptococcus | 0.23% |  | Phoma | 1.07% |
| Neonectria | 0.17% |  | Funneliformis | 0.40% |  | Alatospora | 0.22% |  | Mortierella | 1.00% |
| Alatospora | 0.16% |  | Mortierella | 0.35% |  | Malassezia | 0.19% |  | Tetracladium | 1.00% |
| Vankya | 0.16% |  | Hymenoscyphus | 0.30% |  | Elaphomyces | 0.18% |  | Articulospora | 0.77% |
| Rhodotorula | 0.14% |  | Alternaria | 0.29% |  | Archaeorhizomyces | 0.17% |  | Collembolispora | 0.67% |
| Archaeorhizomyces | 0.13% |  | Leohumicola | 0.26% |  | Leptodontidium | 0.17% |  | Entophlyctis | 0.62% |
| Funneliformis | 0.13% |  | Schizophyllum | 0.26% |  | Rhizophydium | 0.17% |  | Didymella | 0.54% |
| Cadophora | 0.10% |  | Malassezia | 0.22% |  | Pseudogymnoascus | 0.15% |  | Cryptococcus | 0.47% |
| Sebacina | 0.10% |  | Cladosporium | 0.16% |  | Rhizophlyctis | 0.12% |  | Sporobolomyces | 0.47% |
| Beauveria | 0.07% |  | Candida | 0.14% |  | Didymella | 0.11% |  | Candelariella | 0.43% |
| Exophiala | 0.07% |  | Pseudogymnoascus | 0.14% |  | Collembolispora | 0.10% |  | Funneliformis | 0.42% |
| Leucostoma | 0.07% |  | Xenostigmina | 0.13% |  | Tuber | 0.10% |  | Entoloma | 0.37% |
| Entoleuca | 0.05% |  | Dissoconium | 0.13% |  | Beauveria | 0.10% |  | Cyphellophora | 0.32% |
| Microdochium | 0.05% |  | Saitoella | 0.05% |  | Sebacina | 0.10% |  | Diversispora | 0.31% |
| Mucor | 0.05% |  | Herpotrichia | 0.04% |  | Saitoella | 0.07% |  | Archaeorhizomyces | 0.28% |
| Ramariopsis | 0.05% |  | Entophlyctis | 0.03% |  | Pseudocercospora | 0.07% |  | Rhizophydium | 0.27% |
| Rhizophydium | 0.05% |  | Truncatella | 0.02% |  | Capronia | 0.06% |  | Agonimia | 0.24% |
| Articulospora | 0.03% |  | \ | \ |  | Xylodon | 0.06% |  | Phaeosphaeria | 0.24% |
| Collembolispora | 0.03% |  | \ | \ |  | Coniothyrium | 0.05% |  | Basidiobolus | 0.21% |
| Cryptococcus | 0.03% |  | \ | \ |  | Gyoerffyella | 0.05% |  | Ramariopsis | 0.20% |
| Pezizella | 0.03% |  | \ | \ |  | Bensingtonia | 0.03% |  | Dioszegia | 0.18% |
| Tuber | 0.03% |  | \ | \ |  | Chaetosphaeria | 0.03% |  | Herpotrichia | 0.16% |
| Entophlyctis | 0.02% |  | \ | \ |  | Fusarium | 0.03% |  | Beauveria | 0.15% |
| Herpotrichia | 0.02% |  | \ | \ |  | Meliniomyces | 0.03% |  | Pseudogymnoascus | 0.15% |
| Rhizophlyctis | 0.02% |  | \ | \ |  | Rhodotorula | 0.03% |  | Dactylella | 0.14% |
| Trichoderma | 0.02% |  | \ | \ |  | Hannaella | 0.02% |  | Elaphomyces | 0.14% |
| Basidiobolus | 0.01% |  | \ | \ |  | Sporobolomyces | 0.02% |  | Entrophospora | 0.14% |
| Botryotinia | 0.01% |  | \ | \ |  | Kappamyces | 0.01% |  | Oedogoniomyces | 0.13% |
| Claroideoglomus | 0.01% |  | \ | \ |  | Russula | 0.01% |  | Phaeomollisia | 0.13% |
| Hannaella | 0.01% |  | \ | \ |  | Neonectria | 0.01% |  | Rhinocladiella | 0.13% |
| Hygrophorus | 0.01% |  | \ | \ |  | \ | \ |  | Hymenoscyphus | 0.12% |
| Metacordyceps | 0.01% |  | \ | \ |  | \ | \ |  | Scolecobasidium | 0.12% |
| Mortierella | 0.01% |  | \ | \ |  | \ | \ |  | Coniothyrium | 0.11% |
| Scutellospora | 0.01% |  | \ | \ |  | \ | \ |  | Berkleasmium | 0.10% |
| \ | \ |  | \ | \ |  | \ | \ |  | Rhodotorula | 0.10% |
| \ | \ |  | \ | \ |  | \ | \ |  | Bullera | 0.09% |
| \ | \ |  | \ | \ |  | \ | \ |  | Botryotinia | 0.08% |
| \ | \ |  | \ | \ |  | \ | \ |  | Leptodontidium | 0.08% |
| \ | \ |  | \ | \ |  | \ | \ |  | Myxocephala | 0.08% |
| \ | \ |  | \ | \ |  | \ | \ |  | Coniosporium | 0.07% |
| \ | \ |  | \ | \ |  | \ | \ |  | Gaertneriomyces | 0.07% |
| \ | \ |  | \ | \ |  | \ | \ |  | Rhizophlyctis | 0.07% |
| \ | \ |  | \ | \ |  | \ | \ |  | Ochroconis | 0.06% |
| \ | \ |  | \ | \ |  | \ | \ |  | Physcia | 0.06% |
| \ | \ |  | \ | \ |  | \ | \ |  | Septoria | 0.06% |
| \ | \ |  | \ | \ |  | \ | \ |  | Alatospora | 0.05% |
| \ | \ |  | \ | \ |  | \ | \ |  | Arrhenia | 0.05% |
| \ | \ |  | \ | \ |  | \ | \ |  | Inocybe | 0.05% |
| \ | \ |  | \ | \ |  | \ | \ |  | Magnaporthe | 0.05% |
| \ | \ |  | \ | \ |  | \ | \ |  | Mycena | 0.05% |
| \ | \ |  | \ | \ |  | \ | \ |  | Pezizella | 0.05% |
| \ | \ |  | \ | \ |  | \ | \ |  | Rhynchosporium | 0.05% |
| \ | \ |  | \ | \ |  | \ | \ |  | Rinodina | 0.05% |
| \ | \ |  | \ | \ |  | \ | \ |  | Bilimbia | 0.04% |
| \ | \ |  | \ | \ |  | \ | \ |  | Coprinellus | 0.04% |
| \ | \ |  | \ | \ |  | \ | \ |  | Glarea | 0.04% |
| \ | \ |  | \ | \ |  | \ | \ |  | Leptosphaeria | 0.04% |
| \ | \ |  | \ | \ |  | \ | \ |  | Monilinia | 0.04% |
| \ | \ |  | \ | \ |  | \ | \ |  | Candida | 0.03% |
| \ | \ |  | \ | \ |  | \ | \ |  | Cladosporium | 0.03% |
| \ | \ |  | \ | \ |  | \ | \ |  | Crocicreas | 0.03% |
| \ | \ |  | \ | \ |  | \ | \ |  | Hypocrea | 0.03% |
| \ | \ |  | \ | \ |  | \ | \ |  | Microbotryum | 0.03% |
| \ | \ |  | \ | \ |  | \ | \ |  | Neophaeosphaeria | 0.03% |
| \ | \ |  | \ | \ |  | \ | \ |  | Tremella | 0.03% |
| \ | \ |  | \ | \ |  | \ | \ |  | Ambispora | 0.02% |
| \ | \ |  | \ | \ |  | \ | \ |  | Chlorociboria | 0.02% |
| \ | \ |  | \ | \ |  | \ | \ |  | Clavulina | 0.02% |
| \ | \ |  | \ | \ |  | \ | \ |  | Hypoderma | 0.02% |
| \ | \ |  | \ | \ |  | \ | \ |  | Kappamyces | 0.02% |
| \ | \ |  | \ | \ |  | \ | \ |  | Lecanora | 0.02% |
| \ | \ |  | \ | \ |  | \ | \ |  | Lophodermium | 0.02% |
| \ | \ |  | \ | \ |  | \ | \ |  | Malassezia | 0.02% |
| \ | \ |  | \ | \ |  | \ | \ |  | Phialocephala | 0.02% |
| \ | \ |  | \ | \ |  | \ | \ |  | Phialophora | 0.02% |
| \ | \ |  | \ | \ |  | \ | \ |  | Pseudocercosporella | 0.02% |
| \ | \ |  | \ | \ |  | \ | \ |  | Raffaelea | 0.02% |
| \ | \ |  | \ | \ |  | \ | \ |  | Schizangiella | 0.02% |
| \ | \ |  | \ | \ |  | \ | \ |  | Tuber | 0.02% |
| \ | \ |  | \ | \ |  | \ | \ |  | Wickerhamomyces | 0.02% |
| \ | \ |  | \ | \ |  | \ | \ |  | Zasmidium | 0.02% |
| \ | \ |  | \ | \ |  | \ | \ |  | Amanita | 0.01% |
| \ | \ |  | \ | \ |  | \ | \ |  | Caloplaca | 0.01% |
| \ | \ |  | \ | \ |  | \ | \ |  | Chaenotheca | 0.01% |
| \ | \ |  | \ | \ |  | \ | \ |  | Exidia | 0.01% |
| \ | \ |  | \ | \ |  | \ | \ |  | Gibberella | 0.01% |
| \ | \ |  | \ | \ |  | \ | \ |  | Hyphodontia | 0.01% |
| \ | \ |  | \ | \ |  | \ | \ |  | Lentamyces | 0.01% |
| \ | \ |  | \ | \ |  | \ | \ |  | Melanoleuca | 0.01% |
| \ | \ |  | \ | \ |  | \ | \ |  | Meliniomyces | 0.01% |
| \ | \ |  | \ | \ |  | \ | \ |  | Mucor | 0.01% |
| \ | \ |  | \ | \ |  | \ | \ |  | Mycosphaerella | 0.01% |
| \ | \ |  | \ | \ |  | \ | \ |  | Ochrolechia | 0.01% |
| \ | \ |  | \ | \ |  | \ | \ |  | Pyrenopeziza | 0.01% |
| \ | \ |  | \ | \ |  | \ | \ |  | Rhizopus | 0.01% |
| \ | \ |  | \ | \ |  | \ | \ |  | Strelitziana | 0.01% |
| \ | \ |  | \ | \ |  | \ | \ |  | Subplenodomus | 0.01% |
| \ | \ |  | \ | \ |  | \ | \ |  | Utharomyces | 0.01% |
| \ | \ |  | \ | \ |  | \ | \ |  | Vankya | 0.01% |
